# Rheology of Aqueous Solutions in the Presence of Proton Exchange Membrane: Surface Tension

**DOI:** 10.3390/polym18010036

**Published:** 2025-12-23

**Authors:** Svetlana L. Timchenko, Yurii Yu. Infimovskii, Evgenii N. Zadorozhnyi, Nikolai A. Zadorozhnyi

**Affiliations:** Department of Fundamental Sciences, Bauman Moscow State Technical University, 2-nd Baumanskaya Street 5, Building 1, 105005 Moscow, Russia; inf-yura@yandex.ru (Y.Y.I.); eugenezador@gmail.com (E.N.Z.); nikazador@mail.ru (N.A.Z.)

**Keywords:** Nafion, distilled water, methylene blue, ascorbic acid, surface tension, diffusion, membrane soaking, dissociation zone, membrane-solution interface

## Abstract

Controlling the rheological properties of liquids allows for the regulation of effective movement, transport of substances, and processes in biological systems. This work presents an experimental investigation into the influence of the proton-exchange polymer membrane Nafion on the surface tension coefficient (STC) of distilled water, aqueous solutions of two methylene blue (MB) forms, and ascorbic acid (AA). Immediately upon membrane immersion in the solutions, a sharp decrease in the surface tension of distilled water, as well as of the oxidized and reduced forms of MB, occurs. The observed narrow time interval is associated with the formation of an exclusion zone near the membrane–solution interface, containing dissociated sulfonate groups $\left(SO_{3}^{-}\right)$. The value of the time interval depends on the type of aqueous solution. At long soaking of the membrane in solutions, we obtained: for the aqueous solution of Mb^+^ (blue-coloured solution) the STC value eventually increases by about 5%, and for the reduced form of methylene blue MbH^0^-colourless solution, the STC value decreases by 4%. The STC value of the solutions formed during diffusion into the membrane has a significantly lower value compared to the STC of distilled water by 20% for the Mb^+^ form and by 24% for the MbH^0^ form of MB. The presence of the membrane in the aqueous AA solution causes only an increase in the STC value of the solution. Ultimately, for the solution with a concentration of 5 g/L, this increase reached 15% relative to the STC value of the original AA solution. The change in surface tension of the investigated solutions in the presence of the membrane is due to their adsorption onto the membrane surface. Fourier-transform infrared (FTIR) spectroscopy investigation of distilled water, MB, and AA solution diffusion into the membrane across the range (370–7800) cm^−1^ confirms the process nonlinearity and enables identification of distinct time intervals corresponding to membrane swelling stages. The positions of IR transmission minima for membranes containing water and solution components remain unchanged; only the numerical values of the transmission coefficients vary. Using spectrophotometry, absorption lines of the membrane with adsorbed components of MB and AA solutions were identified in the range of (190–900) nm. The absorption spectra of dried membranes with adsorbed Mb^+^ and AA solutions show a redshift to the IR region for the Nafion with Mb^+^ and a shift to the UV region for the Nafion soaked in an aqueous ascorbic acid solution. A surface tension gradient at the membrane–solution interface can induce concentration-capillary convection in the liquid.

## 1. Introduction

Polymer membranes based on perfluorinated sulfonic acid have been extensively studied over the past few decades [1,2,3,4] and have found applications in humidity control systems, as well as a key role in fuel cell and hydrogen energy technologies [5]. Perfluorinated membranes have high chemical and thermal resistance due to their C–F bond strength. This makes them highly selective for cation transfer and have low electrical resistance. Additionally, such membranes have good mechanical strength, making them durable and long-lasting. Membranes based on perfluorinated polymer due to the introduction of perfluorovinyl ether groups ending in sulfonic groups are ion-exchange membranes and have a special structure and morphology [1]. Tetrafluoroethylene forms their base, provides mechanical strength and stability, and is a hydrophobic part of such membranes. The hydrophilic part of the polymer is formed by sulfonic groups, which provide the polymer with ionic conductivity. The presence of hydrophilic sulfonic groups and hydrophobic perfluorinated chains in the membrane ensures the formation of a system of pores connected by narrow channels. In this case, the efficiency of ion transport of the membrane is determined by the concentration of ionactive side groups [1,3,4].

Among the ion-exchange membranes, the Nafion membrane should be distinguished. Nafion is a sulfonated fluoropolymer based on tetrafluoroethylene [2,4]. Nafion is produced by copolymerization of a comonomer of perfluorinated vinyl ether with tetrafluoroethylene and consists of groups of perfluorovinyl ether completed with sulfonic groups (${SO}_{3}^{-}$) based on tetrafluoroethylene. Tetrafluoroethylene is a highly hydrophobic matrix, and the sulfone groups are hydrophilic compounds [1,2,3,4]. The chemical formula of the Nafion proton exchange membrane is C_7_HF_13_O_5_S·C_2_F_4_ [1,2,4].

So, the Nafion membrane is a heterogeneous system in which hydrophobic perfluorinated chains provide rigidity and mechanical strength of the membrane, and the hydrophilic part provides proton transfer [1,3,6]. When the Nafion membrane is immersed in water, the anionic groups of the membrane are hydrated and form a continuous network of hydrogen bonds. Since the Nafion membrane has a sufficiently high conductivity with relation to protons, it is called a proton exchange membrane. When the Nafion membrane is immersed in water, the anionic groups of the membrane hydrate and form a continuous network of hydrogen bonds [4].

In work [7], the proton transport mechanisms and conductivity optimization are discussed in detail. In work [8], neutron reflectometry revealed that lamellar structures exist at the interface between SiO_2_ and hydrated Nafion film, consisting of thin alternating layers with water-rich regions adjacent to Nafion. The observed interfacial structures affect the performance, reliability, and optimization of proton-exchange membranes in fuel cells and membrane-electrode assemblies.

The Nafion membrane is the first of a class of synthetic polymers that have ion-exchange properties [9,10,11,12]. In article [9], the authors obtained rectified current-voltage (I–V) characteristics at varying concentrations of sodium-ion electrolyte during transport through both a micro-hole and a cation-exchange membrane. The data from reference [9] demonstrate a linear current-concentration dependence, indicating controlled electro migration.

Work [13] demonstrated that interfacial electron transfer kinetics become retarded when using a fluorinated SAM (a self-assembled monolayer) instead of the conventional Au/Nafion electrode configuration.

The properties of Nafion membranes in aqueous solutions are determined by the polymer’s structure and the presence of ion-active side chains containing sulfonic groups ${SO}_{3}^{-}$, as thoroughly investigated in studies [14,15,16,17,18]. Water-swollen Nafion exhibits what is known as amphiphilic character [1,2,4,14,16]. The Nafion membrane develops an internal structure of cylindrical micelles with water-filled channels measuring 2–3 nm in diameter. At the interface of these channels, terminal sulfonic groups dissociate according to the scheme: $R-SO_{3}H+H_{2}O↔R-{SO}_{3}^{-}+H_{3}O^{+}$ and the inner channel surfaces become negatively charged. As a result, cations are effectively drawn into the channels [1,2,16], a property exploited for spatial separation of $H^{+}$ and ${OH}^{-}$ ions in hydrogen energy systems. As it was determined earlier, the proton conductivity of the membrane increases with an increase in water absorption during hydration [8]. Along with this, it has been experimentally proven that the permeability of the Nafion membrane to water increases with increasing temperature [18].

The interfacial water structure at Nafion membrane surfaces has been characterized in references [4,12,14]. It was found that, when the membrane was immersed in an aqueous suspension of colloidal microspheres [14], the colloidal microspheres were repelled from the membrane surface. Effective repulsion is observed at distances up to several hundred micrometers from the membrane surface. This phenomenon is known as the ‘exclusion zone’ (EZ). The domain occupied by the disentangled polymer fibers exhibits dimensions similar to those of the exclusion zone characterized in references [11,12,16].

The effect of UV irradiation (266 and 369 nm) in the geometry of sliding incidence on the surface of a Nafion membrane soaked in water was investigated in [15]. The photoluminescence signal of a polymer is sensitive to the degree of its water impregnation and does not monotonously depend on the isotopic composition.

In [12], the Nafion membrane (N117) was clamped between the glasses of a liquid cuvette, which made it possible to observe the effect of mechanical stresses arising in its hydrophobic framework and the subsequent dissociation of sulfogroups. After filling a liquid cuvette with $CaF_{2}$ glasses with water, the formation of a liquid-free cavity was observed. The resulting cavity eventually collapses, and the Nafion membrane is completely covered with a layer of liquid. At this moment, the polymer is impregnated with water under conditions similar to those realized in a Petri dish, and the membrane is completely immersed in water. If the membrane is located in a Petri dish in which a sufficient amount of water is poured, then we do not see the effect of the cavities collapsing. These cavities arise due to the deformation of the hydrophobic framework clamped between the cell windows. The disappearance (collapse) of cavities is caused by the subsequent dissociation of ion-active membrane side chains containing sulfogroups ${SO}_{3}^{-}.$ Initially, the hydrophobic Nafion, after the dissociation of sulfogroups, becomes hydrophilic, filled with water, and its framework straightens out. Also, in [12], the wetting angle from a drop of water applied to the surface of the Nafion was measured as it was soaked in water. An abrupt decrease in the wetting angle was found, which indicates the transition of the membrane from a hydrophobic to a hydrophilic state.

The review [16] describes in detail the nanostructure of the polymer matrix of the Nafion membrane, which is formed when it swells in aqueous solutions. The nanostructure of the polymer consists of conducting cylindrical channels, which are similar to the microemulsion of the reverse phase [19,20,21]. The microstructure of bicontinual microemulsions consists of aqueous tubes coated with surfactant molecules [19,22]. The variability of the self-organizing structure of Nafion is due to two effective forces: the powerful influence of the packaging of the hydrophobic membrane framework and the intersurface molecular forces that manifest themselves due to sulfogrups. The size of the excluded zone and the mobility of the membrane skeleton links are influenced by the chirality and isotopic composition of the aqueous base, as shown in [23,24].

The process of proton transfer in solution by means of a Nafion is a combination of two mechanisms, the dominance of which depends on the degree of hydration of sulfogroups [3,6,16]. The first mechanism is structural diffusion, known as the Grotthuss mechanism. This mechanism dominates at high hydration levels (λ > 5–6), where λ represents the number of water molecules ($H_{2}O$) per sulfonate group $\left(SO_{3}^{-}\right)$. In this process, proton transport occurs via the breaking and forming of O-H bonds within the extended hydrogen-bonded water network inside the membrane channels. The sulfonic acid groups remain predominantly in a dissociated state, $SO_{3}^{-}+H^{+}\to SO_{3}H$, providing the source protons. Channels filled with water form inside the membrane. The proton, overcoming the potential barrier, tunnels from the hydroxonium ion ${H_{3}O}^{+}$ to the neighboring $H_{2}O$ molecule through an oriented network of hydrogen bonds. The second mechanism, called the Vehicle mechanism, occurs at a low hydration level. In this mechanism, proton transfer is facilitated by the physical diffusion of larger ions, such as the hydronium ion (${H_{3}O}^{+}$). In practice, factors such as temperature, hydration level, and membrane morphology determine the dominance of one proton transport mechanism over the other.

The ion-exchange properties of membranes depend on their production method. Membranes produced by extrusion casting have a higher diffusion rate compared to membranes produced by injection molding. The size of the membrane side chains also affects the diffusion rate: shorter side chains ensure faster diffusion [25]. NMR diffusiometry in a static magnetic gradient field was used to analyze the effect of water content, as well as membrane thickness and side chain length, on the diffusion coefficient [25]. A decrease in the diffusion coefficient, observed with reduced temperature or lower water content, attenuates proton transport mechanisms. In this case, the role of water molecules bound to membrane pore walls in facilitating proton transfer becomes significantly enhanced. Water confined within the nanochannels of perfluorosulfonic acid (PFSA) ion-exchange membranes can be considered as existing in two distinct states: surface-adsorbed water and bulk-like water in the central part of the membrane. Water in these states is characterized by different activation energies of diffusion and conduction [25].

The review [26] presents experimental methods for studying the properties of perfluorosulfonic acid (PFSA) membranes and discusses the transfer phenomena, performance characteristics, and modes of membrane destruction.

Research on the use of membranes is relevant not only in hydrogen production systems. For example, research on the interaction of the Nafion polymer membrane with aqueous solutions of various forms of MB and with ascorbic acid is relevant from an applied medical point of view, since these solutions exhibit an antiviral effect and are used in the fight against COVID-19 [27,28,29]. In oncology, methylene blue is recognized as a potent photosensitizer. Under light activation, MB exhibits strong antiviral activity and promotes destruction of pathological cells [27].

In [30], the forms of MB and UV-controlled triplet photochemistry of MB are described in detail. It has been demonstrated that MB undergoes photooxidative quenching by dissolved oxygen. This property of MB is utilized in photodynamic therapy [31,32]. The authors of [33] found that MB promotes NADH (Nicotinamide adenine dinucleotide plus hydrogen) oxidation, increases the level of NAD^+^ (Nicotinamide adenine dinucleotide plus) and the activity of sirtuin 3 deacetylase, and also reduces the level of lysine acetylation in the mitochondria of the heart in diabetes. NADH is the reduced form of nicotinamide adenine dinucleotide (NAD). NAD^+^ is the oxidized form of NAD. NADH is a coenzyme, that is, a compound that helps other compounds called enzymes to carry out chemical reactions in the body. NAD^+^ is present in every living cell of the body and participates in metabolic processes, providing cellular bioenergetics. NADH supplements can be useful for reducing energy in the body, heart disease, decreased cognitive abilities, and chronic fatigue syndrome in general [34]. Concurrently, MB increases oxygen consumption, reduces glycolysis, and increases glucose uptake in vitro.

In works [35,36], the Nafion matrix was considered as a model medium for further studying the interaction of drugs with a living cell in the treatment of viral diseases. The rate of adsorption of MB on the surface of the Nafion polymer membrane was studied in near-UV and visible-light spectrophotometry experiments [35]. It was found that the rate of MB adsorption is controlled by the isotopic composition of the MB solution. If the deuterium content is 157 ppm, then the adsorption rate of MB is about 3 times lower than in the case of a deuterium content of 3 ppm. The water desorption rate from the membrane surface during drying is also three times higher for the MB solution with 157 ppm deuterium content. This suggests that these effects are associated with slowed diffusion processes within the layer of unwound Nafion polymer fibers [12,15,16].

The adsorption process of MB and AA into Nafion membranes from aqueous solutions prepared with bidistilled water was investigated using IR Fourier spectrometry (FTIR) in the range of (370–7800) cm^−1^ and by spectrophotometry in the UV and visible ranges (190–900 nm) [36]. Upon polymer swelling in the test solutions, changes in membrane color and infrared transmittance were observed, indicating chemical reactions occurring at the membrane–solution interface. The «Nafion–MB» system exhibits metachromasia. The dehydrated membrane incorporating reduced MB in form MbH^0^ exhibited chromatic transition from white to blue coloration. It is logical to assume that a redox reaction occurred in the membrane containing McH^0^ during its prolonged drying, which is a manifestation of its proton-exchange properties. As a result, the leukoform MbH^0^ was oxidized to the initial state of Mb^+^. The Nafion membrane, due to its high cationic conductivity, promotes proton transfer and thereby will affect the rate of redox reactions, in particular, reactions involving MB.

Low-frequency harmonic vibrations (7–30) Hz also have an effect on the properties of the aqueous MB solution [37]. It was found that when low-frequency harmonic vibrations are applied to the solution, the MB reduction reaction is reversible, and chemical compounds, semiquinone radicals, formed at intermediate stages of the redox sonochemical process, were identified by EPR spectroscopy.

The formation of an exclusive zone near the Nafion membrane, which has hydrophilic properties, was investigated by Gerald H. Pollack and colleagues [11,38,39]. The so-called “exclusion zones” and exclusive zones formed near the surface of the membrane with water have a more orderly phase than the main water, with local charge separation between exclusion zones and regions beyond them. This is confirmed by measurements of the pH of solutions, nuclear magnetic resonance, luminescence, and IR spectroscopy. The near-surface exclusion zone expands significantly in the presence of incident radiant energy [11]. Interfacial water differs from bulk water in mechanical and electrical properties. Nonproliferation zones have a negative electrical potential of the order of −100 mV compared to bulk water, with a corresponding distribution of positive protons in the bulk water area outside the nonproliferation zone [39]. In practice, we have a situation where the interphase phase includes solutes. In this case, it is necessary to create a mechanism that allows the interaction of the solute with the surface, including between substrates and enzymes [40]. This is important in medicine and biology. Therefore, studies of the properties of solutions near the membrane surface are relevant.

Interphase mass transfer near an ion exchange membrane differs from bulk transfer in the volume of water and aqueous solutions. For efficient water transportation, it is necessary to increase the proton conductivity for protons in fuel cell proton exchange membranes (PEMs) as much as possible. The correlation between interphase water transport and proton conductivity was established using coherent Raman spectroscopy and measurements of through-channel proton conductivity [41]. Practically all proton exchange membranes are characterized by the fact that the total diffusion capacity of water can be represented as a linear combination of the diffusion capacity of bulk and interfacial water. However, the diffusion capacity of interfacial water is consistently higher than that of bulk water.

As can be seen from a short review of experimental works related to the Nafion membrane, most of them investigate the internal structure of the polymer, and in this work the properties of aqueous solutions in which the Nafion membrane was located are investigated. The research on the rheology of aqueous solutions near the surface of the proton exchange membrane will add information about its properties in the presence of aqueous solutions and will explain the features of the diffusion of aqueous solutions into the membrane and microfluidics of the subsurface layer.

The properties of the surface layer of the membrane in aqueous solutions are important not only from the point of view of its application in fuel systems, but also from the point of view of the exchange processes formed near the polymer surface in adsorption systems. In recent works the effect of the membrane on the crystallization of salts and acids from supersaturated aqueous solutions has been investigated [42,43]. However, the studies did not consider the influence of the rheology of solutions on the characteristics of crystal growth. In our work, we show the relevance of accounting, in particular, the STC of solutions. Because the size of the crystallization nucleus and the rate of crystallization depend on the coefficient of surface tension of the solutions.

In this paper, we show the importance of accounting, in particular, the STC of aqueous solutions. We assume that the level of hydration depends on the type of aqueous solution. Thus, the diffusion coefficient of protons and cations will vary. Therefore, we tried to study the contribution of the Nafion membrane interface to the surface tension of aqueous solutions of MB and AA. This approach will enable future determination of the membrane’s effect on the kinetics of redox and diffusion processes. The diffusion of MB and AA into the membrane may allow the redox reaction to occur directly in the membrane. This work investigated the influence of the polymer membrane’s ion-exchange properties on the surface tension coefficient of an aqueous solution of the oxidized form MB–Mb^+^, a reduced form MB—a white solution—MbH^0^ and an aqueous solution of ascorbic acid. Using spectrometry methods in the UV, visible, and IR ranges, characteristic transmission/absorption lines of the Nafion membrane were obtained when it was soaked in the studied solutions.

## 2. Materials and Methods

The properties of the Nafion membrane during its soaking in distilled water, MB and AA solutions were investigated using IR Fourier spectroscopy, UV–Vis absorption spectroscopy, and surface tension measurements at the membrane–solution interface. Measurements of the surface tension coefficient of distilled water, aqueous solutions of MB and AA were carried out directly near the surface of the membrane during its soaking.

All solutions were prepared using distilled water—pH = 7.05. The distilled water was obtained using a Milli-Q system (Merck KGaA, Darmstadt, Germany). The deuterium content in the water was (157 ± 1) ppm. To prepare a solution of Mb^+^ and AA methylene blue powder and ascorbic acid were used. Ultrapure MC and AA powder was purchased from Macsen Labs (N.K. Agrawal Group, Udaipur, Rajasthan, India). The basic Mb^+^ solution (blue solution) has a spectrum with absorption maxima for the dimer λ = 615 nm and the monomer λ = 665 nm [25,30,37]. To prepare the aqueous ascorbic acid solution, 0.1 g of ascorbic acid powder was dissolved in 20 mL of distilled water. The reduced form (LMB—Leuco form of methylene blue) of MB was obtained as a result of the reaction of a solution of Mb^+^ with a solution of AA:

Mb^+^ + 2e^−^ + H^+^ = MbH^0^ (1)

Plates of Nafion polymer N117 (Sigma Aldrich, St. Louis, MO, USA) with a thickness of L_0_ = 175 microns and an area of 2 × 3 cm^2^ were soaked in these solutions. The pH values of the initial solutions were: Mb^+^—pH 5.41; AA—pH 3.42.

The surface tension of distilled water and aqueous solutions of MB and AA was measured directly adjacent to the membrane surface during soaking. Two series of experiments were performed. Two series of experiments were performed. The first series is the measurement of the STC of the initial solutions; the second series is the measurement of the STC of solutions during the soaking of the Nafion membrane. The experiments were carried out at a solution temperature of T = 20 °C.

**Figure 1:**
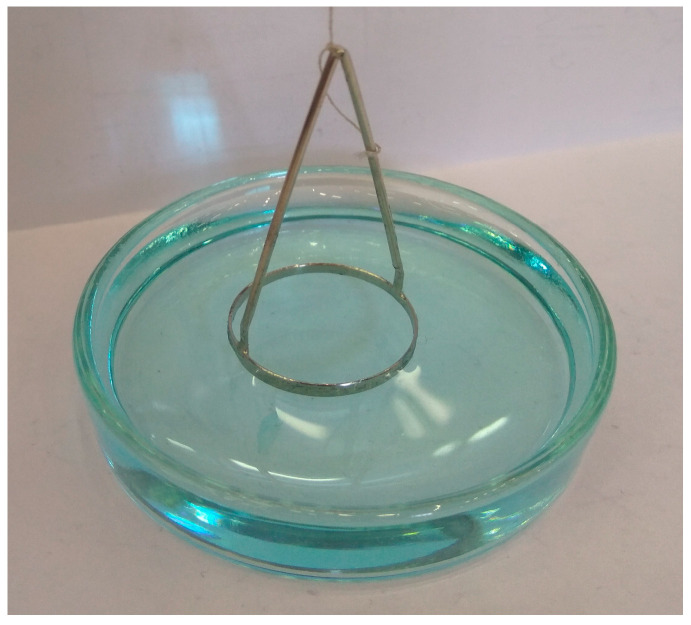
Measurement of the STC of the solutions using the Du Noüy method.

The solutions were alternately poured into a Petri dish. The volume of the solution for measuring STC was V = 12 mL. Before immersion of the membrane in the solution, the STC of the initial solution was measured. A membrane with dimensions of 2 × 3 cm^2^ and 175 μm thickness was then placed in the solution. Measurements of the STC of solutions with a membrane were carried out immediately after immersion of the membrane in solutions with an interval of about 1 min. The ring detachment method—the Du Noüy method (Phywe, Göttingen, Germany)—using a metal (steel) ring was chosen for measuring the solution STC. Figure 1 shows the Petri dish with the test solution. During the measurement, the ring was located near the membrane surface, which made it possible to measure the surface tension of the liquid near the membrane surface.

The formula was used to calculate the STC value of the solutions:

(2) $$\sigma =\;\frac{F_{\sigma }}{2\pi \left(R_{1}+\;R_{2}\right)}=\;\frac{F_{\sigma }}{\pi \left(D_{1}+\;D_{2}\right)},$$

where $F_{\sigma }$ is the ring separation force, mN; the diameters of the inner and outer surfaces of the ring are equal, accordingly, —D_1_ = 19.1 mm—D_2_ = 20 mm.

The instrumental measurement error of the STC solutions was 0.05 mN/m. The number of measurements of the STC values of solutions in a specific time interval was 3. Repeated measurements of kinetic dependences were performed at least 5 times at a constant temperature (20 °C) and humidity in the room (60%). Measurements taken when the system is in an equilibrium state have lower error than those taken during kinetic (non-equilibrium) measurements.

The adsorption kinetics of the test solutions into the membrane were detected by spectrophotometry. For this purpose, the absorption coefficient of the membranes was measured during their soaking in the solutions. The membrane absorption spectra were recorded using a spectrophotometer PB 2201 («SOLAR», Minsk, Belarus) in the (190–800) nm range with a resolution of 2 nm.

IR transmission spectra of solutions and membranes were acquired using an analytical FTIR spectrometer—FSM 2201 (INFRASPEK Ltd., Saint Petersburg, Russia) operating in the wavenumber range (370–7800) cm^−1^ with a nominal resolution of ≤1 cm^−1^. The absolute wavenumber accuracy was ±0.05 cm^−1^. The membrane samples were immersed in a Petri dish containing distilled water and the test solutions, and the transmission spectrum of the membrane was recorded during the soaking process.

## 3. Results

**Figure 2:**
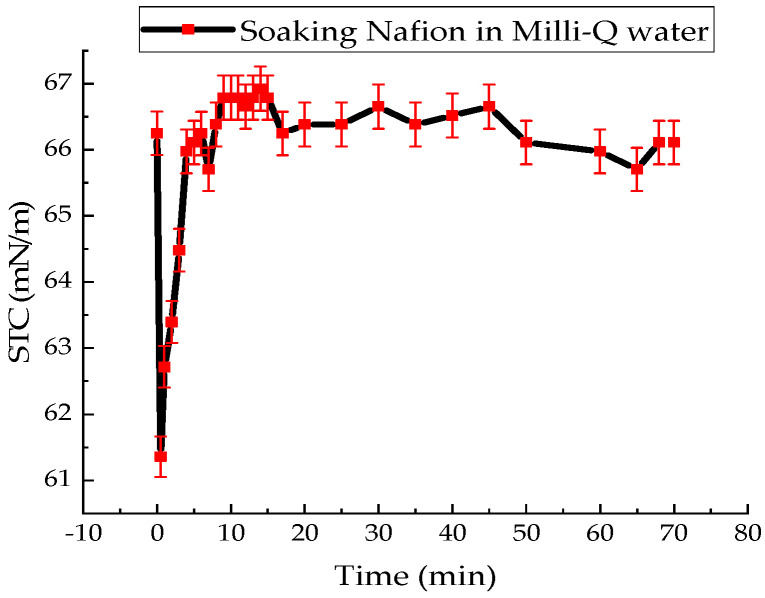
Time dependence of the surface tension coefficient of distilled water during Nafion soaking.

The value of the surface tension coefficient of distilled water (MQ, pH = 7.05, solution temperature T = 20 °C) without Nafion was measured at 71.68 ± 1.43 mN/m. The kinetic profile of STC changes for distilled water containing an immersed Nafion is shown in Figure 2.

**Figure 3:**
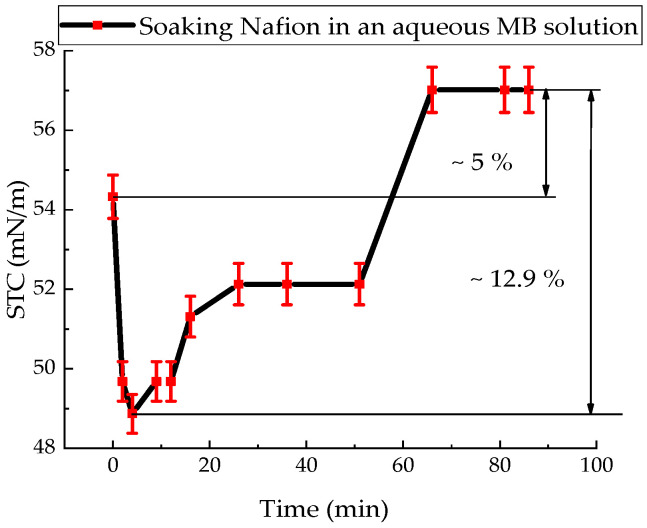
Time dependence of the surface tension coefficient of an aqueous Mb^+^ solution during Nafion membrane soaking.

The STC value of the aqueous solution of the oxidized form MB (Mb^+^) was (54.30 ± 1.10) mN/m. The kinetics of the change in the STC value of an aqueous solution of Mb^+^ after immersion of the Nafion in a Petri dish with a solution of Mb^+^ is shown in Figure 3.

**Figure 4:**
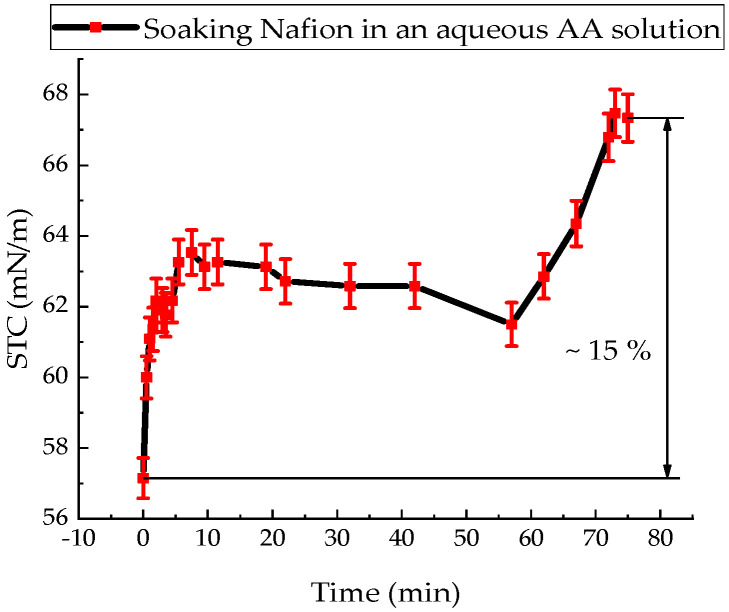
Time dependence of the surface tension coefficient of an aqueous ascorbic acid solution during Nafion membrane soaking.

The value of the STC of an aqueous solution of AA at a temperature of 20 °C was (57.20 ± 1.10) mN/m. Figure 4 shows the surface tension change kinetics of an aqueous ascorbic acid solution upon immersion of a Nafion membrane.

**Figure 5:**
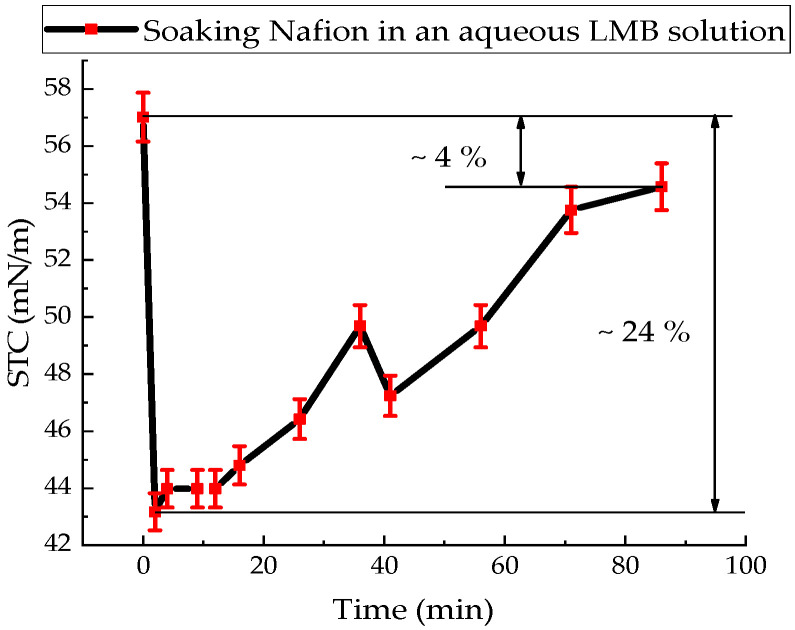
Time dependence of the surface tension coefficient of an aqueous leuco-methylene blue (MbH^0^) solution during Nafion membrane soaking.

The STC value of the aqueous solution of the reduced MB–MbH^0^ form was (57.02 ± 1.20) mN/m. The kinetics of the change in the STC value of an aqueous solution of MbH^0^ after immersion of the Nafion in a Petri dish with a solution of MbH^0^ is shown in Figure 5.

**Figure 6:**
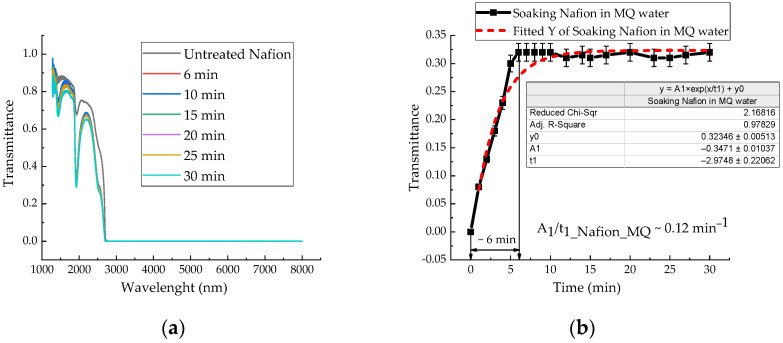
(**a**) The membrane IR transmission spectra during soaking in distilled water. (**b**) Time dependence of the transmittance coefficient of a Nafion membrane during its soaking in distilled water at a wavelength of 1932 nm.

Figure 6a shows the IR transmission spectra of the membrane during soaking in distilled water, recorded at 6, 10, 15, 20, 25, and 30 min, covering the wavelength range from 1282 to 8000 nm. At a wavelength of ~1932 nm, there is a minimum transmission of the untreated Nafion membrane and the membrane during its swelling in water. The transmission coefficient of distilled water also exhibits a minimum at a wavelength of 1932 nm with a value of 0.03, while for an untreated Nafion membrane it is 0.67841—approximately 23 times higher. As the membrane swells, the value corresponding to the minimum transmission coefficient decreases. Figure 6b shows the time dependence of the transmittance at the characteristic minimum (~1932 nm) for a Nafion membrane during soaking in distilled water. This kinetic curve (Figure 6b) was derived from the time-resolved IR transmission spectra in Figure 6a. The significantly higher temporal resolution of the data in Figure 6b allows for a more detailed analysis of the kinetics of distilled water diffusion into the membrane channels. The water uptake (hydration) of the Nafion membrane was estimated from the difference in the IR transmission spectra between the untreated membrane $\left(I_{Nafion}(t)\right)$ and the membrane soaked in water $\left(I_{Nafion+MQ}(t)\right)$ (Figure 6a).

**Figure 7:**
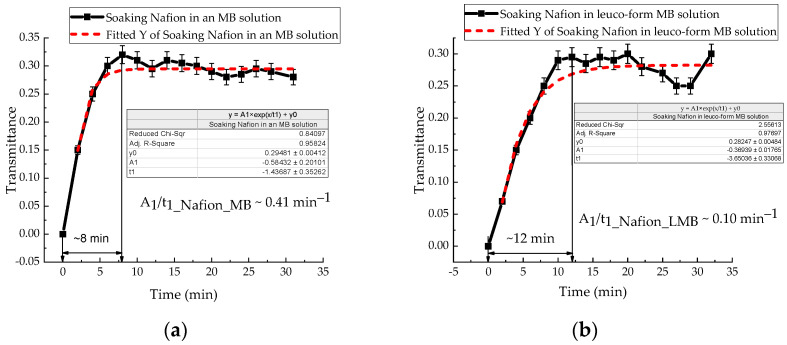
Time dependence of Nafion transmittance during its soaking in aqueous Mb^+^ (**a**) and MbH^0^ (**b**) solutions.

**Figure 8:**
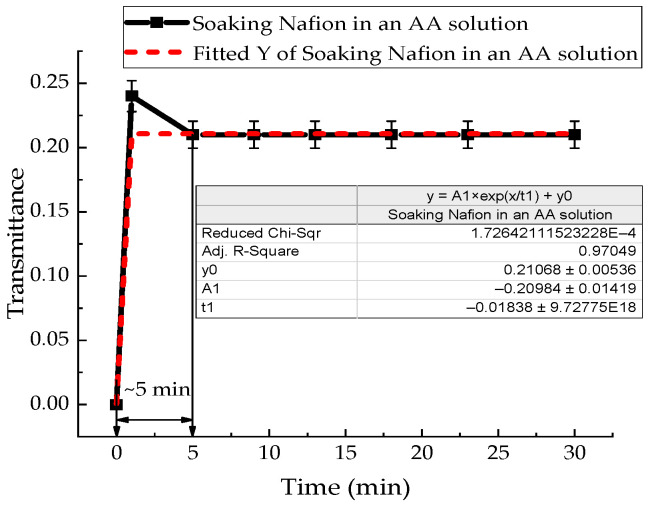
Time dependence of Nafion transmittance during its soaking in an aqueous ascorbic acid solution.

The same experimental procedure was used to investigate the soaking kinetics of the Nafion membrane in Mb^+^, ascorbic acid, and leuco-methylene blue solutions. The IR transmission spectra of Mb^+^, AA, and leuco-MB solutions also exhibit a transmission minimum at a wavelength of ~1932 nm. The absorption kinetics of the investigated aqueous solutions by the membrane are presented in Figure 7a,b and Figure 8. In Figure 6a, Figure 7a,b and Figure 8, a time interval is highlighted that corresponds to the maximum change in the transmission coefficient for the membrane soaked in the test solutions: 6 min for Nafion in distilled water; 8 min for Nafion in MB solution (Mb^+^); 12 min for Nafion in leuco-MB solution; and 5 min for Nafion in AA solution. These time intervals do not represent a state of maximum membrane saturation but rather indicate a period of active ingress for both distilled water and the test solutions into the membrane.

**Figure 9:**
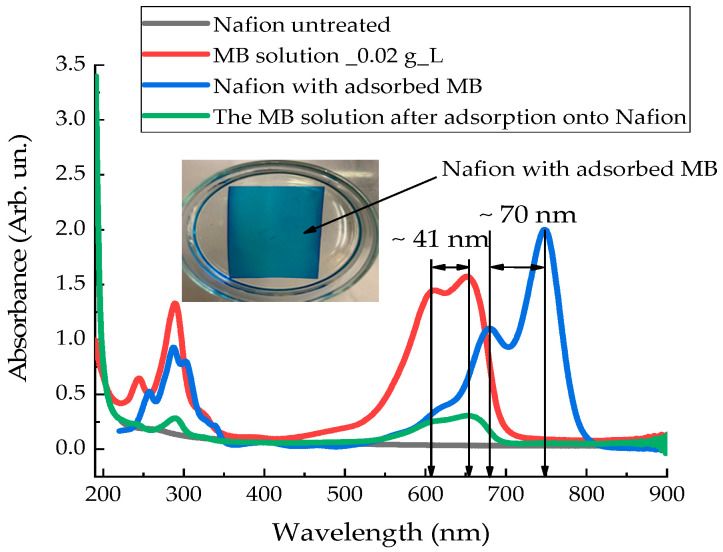
Absorption spectra of untreated Nafion membrane (black curve); aqueous Mb^+^ solution (red curve); Mb^+^ solution after Nafion membrane soaking (green curve); and dried Nafion membrane with adsorbed MB (blue curve).

Figure 9 presents the absorption spectra of the untreated Nafion membrane (black curve), the initial Mb^+^ solution (red curve), the Mb^+^ solution after soaking the Nafion membrane in this solution (green curve), and the dried Nafion membrane with adsorbed Mb^+^ solution (blue curve), recorded using a spectrophotometer (SOLAR) in the (190–800) nm range. The inset to Figure 9 shows a photograph that demonstrates the process of MB adsorption into Nafion—the color of the membrane corresponds to the color of the initial solution, and the solution formed after diffusion is white.

**Figure 10:**
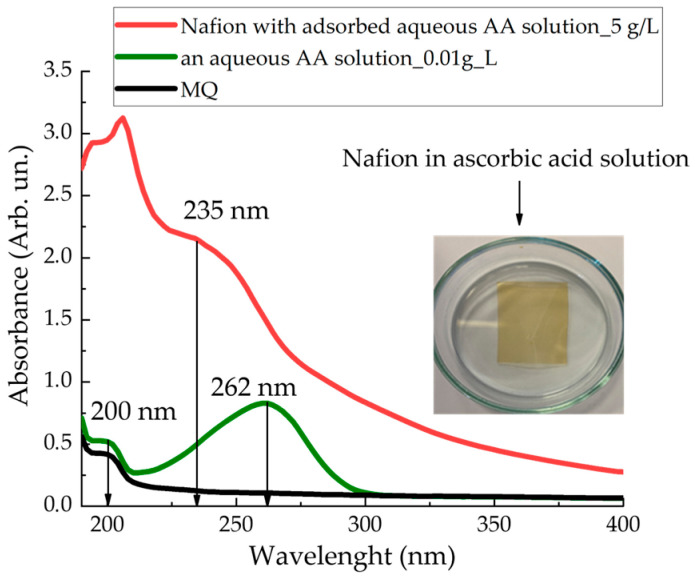
Absorption spectra of the membrane after adsorption from an AA solution (red curve); the aqueous AA solution at a concentration of 0.01 g/L (green curve); and distilled water (black curve).

Figure 10 presents the absorption spectra (190–400) nm of a Nafion membrane after adsorption from an aqueous AA solution (red curve); the aqueous AA solution itself (green curve); and distilled water (black curve). The inset to Figure 10 shows a photograph that illustrates the process of AA adsorption into Nafion. As the AA solution diffuses into the membrane, it acquires a yellowish tint.

An incompletely formed absorption spectral peak for distilled water and the aqueous AA solution appears at a wavelength of ~200 nm. An absorption maximum forms in the absorption spectrum of the Nafion membrane in the wavelength range of ~235 ± 2 nm. This spectral line is close to the absorption line of the initial AA solution, which is observed at 262 nm; however, it is shifted to the UV region by approximately 27 ± 2 nm.

**Figure 11:**
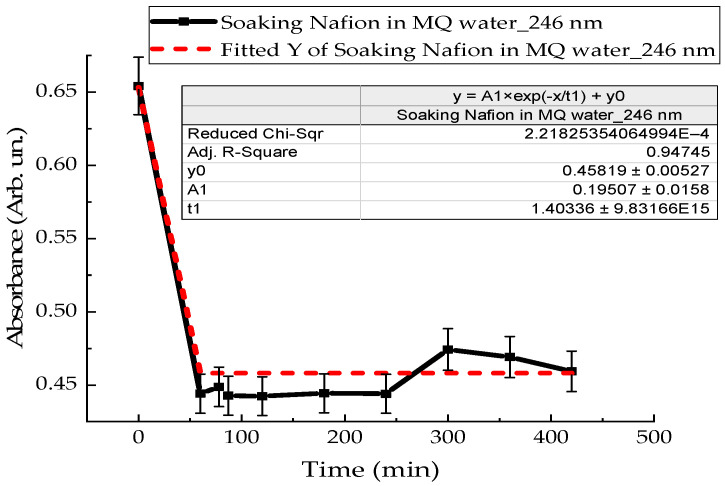
Time dependence of the absorption coefficient of a Nafion membrane during its soaking in distilled water.

**Figure 12:**
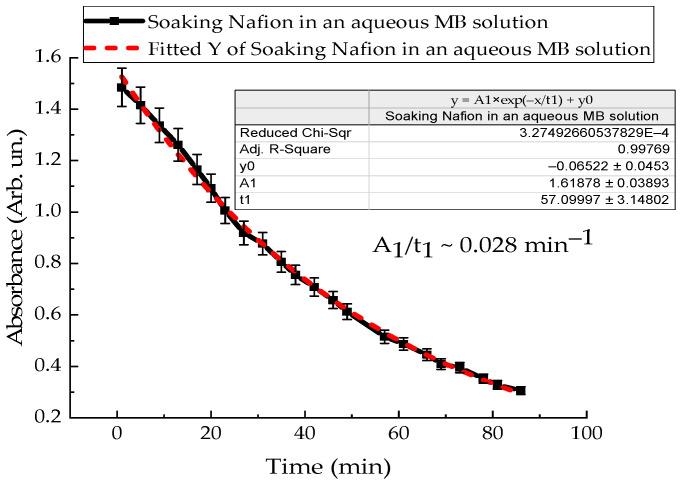
Time dependence of the absorption intensity of the aqueous Mb^+^ solution during Nafion soaking in it.

To clarify the intensity of convection flows near the membrane surface, the kinetics of polymer swelling in distilled water and MB solution were investigated. A Nafion membrane with an area of 3 cm^2^ was placed in a quartz cuvette with distilled water in a volume of 4 mL, and the absorption spectra of Nafion-water were recorded. Figure 11 shows the time dependence of the absorption of distilled water with Nafion at a wavelength of 246 nm. The dependence indicates the rate of Nafion swelling in distilled water (Figure 11). Figure 12 shows the time dependence of the absorption of an aqueous solution of Mb^+^, detected for the MB monomer (652 nm), when soaking Nafion in it. The initial concentration of the MB solution was 0.02 g/L.

## 4. Discussion

Summarizing the results of the experiments, it can be said that the presence of the Nafion membrane in the solution leads to a sharp decrease in the STC value of distilled water and the studied solutions–forms of MB. At the initial stage of soaking Nafion in water, the STC value decreased by an abrupt 7.4% in 30 s. The surface tension of water then returns to its initial value within 300 s (Figure 2). Then there are non-monotonic changes in the water’s STC value relative to its average value, which continue until the end of membrane soaking.

The interval during which the STC value of the Mb^+^ aqueous solution decreases by 10% is 4 min. Then there is a non-monotonic increase in the STC of the Mb^+^ solution to a value of 57.02 mN/m, which lasts for 82 min (Figure 3).

The value of the STC of an aqueous solution of AA in the presence of a membrane only increases (Figure 4). During 86 min of soaking the Nafion in this solution, the value of the STC of the solution increases by 15%. This can be explained by the dominance of the diffusion of AA molecules into the membrane and the formation of stable bonds, compared with the diffusion of water molecules. In [36], by recording the IR transmission spectrum of the membrane in the range (370–7800) cm^−1^, it was found that within a minute from the start of soaking the membrane, the soaking rate of the membrane in the AA solution is 3 times higher than the soaking rate in distilled water.

For an aqueous solution of MbH^0^, an active decrease in the STC value of this solution with membrane is observed within 2 min and reaches 24%. Over the subsequent 84 min, the STC of the MbH^0^ solution with the Nafion membrane increases, ultimately reaching a value approximately 4% lower than the STC of the initial solution (Figure 5).

One of the reasons for the complex kinetics of the STC solutions under study may be the process of adsorption of water and solutions into the membrane. When the membrane is soaked for a long time in solutions: for an aqueous solution of Mb^+^, the STC value eventually increases by about 5%, and for the reduced form of MB, the STC value decreases by 4%. The result can be explained by the process of diffusion of water, oxidized (Mb^+^) and reduced (MbH^0^) forms of MB onto the membrane surface. The value of the STC of the tested solutions formed during diffusion into the membrane is significantly lower than the STC of distilled water by 20% for Mb^+^ and 24% for MbH^0^.

As mentioned earlier [1,2,3,4,16], upon dissociation of sulfone groups, the inner surface of the Nafion channels becomes negatively charged. Cations can effectively enter these channels. In this case, the effective attraction of cations is expressed in diffusion into the membrane of the oxidized form of MB (Mb^+^). Due to the diffusion of aqueous solution Mb^+^ into Nafion, the concentration of the Mb^+^ solution decreases, resulting in an increase in the surface tension of the solution. MB is not a polar molecule. However, in the state of an aqueous solution, it behaves like a cation. Consequently, when the membrane is immersed in the MB solution, a Coulomb interaction will occur between the sulfone groups of the Nafion and MB membranes. Ultimately, during prolonged soaking of the membrane in MB solutions, we obtain a membrane that has additional absorption bands in the (190–900) nm range (Figure 9).

**Figure 13:**
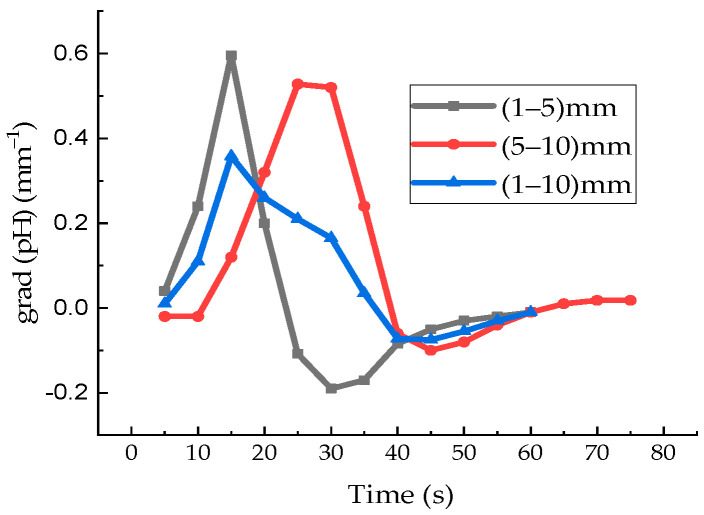
Time dependence of the pH gradient at different distances from the membrane surface: (1–5) mm (black curve); (5–10) mm (red curve); (1–10) mm (blue curve) [constructed based on the results of the work [11]].

The concentration of water in the polymer determines its adsorption capacity, which increases with an increase in the amount of water inside the membrane during swelling [18]. It can be assumed that near the membrane surface, there is an active decrease in the hydrogen index of the solution, pH, as observed in the work for distilled water [11]. Active ion transport occurs in the vicinity of the membrane surface upon its immersion in aqueous solutions. In this case, the contact of two media having a similar pH value, such as Nafion and an aqueous solution of AA, leads to the reactive diffusion of AA into the membrane and, as a result, the value of the STC of the solution increases. An active decrease in the STC value of distilled water and MB solutions, as well as an increase in the STC value of aqueous solution AA, which is observed at the initial stage of membrane soaking, can be explained by transient processes in the system associated with the movement of ions and electrons. The experimental results presented in [11] indicate the imbalance of the «Nafion-water» system and confirm our hypothesis about the occurrence of a zone of dissociation of sulfonic groups and relaxation of the volume charge in aqueous solutions near the surface of the ion exchange membrane. Figure 13 shows the pH gradient of distilled water near the surface of the Nafion membrane.

Figure 13 was constructed based on the results of the authors of [11]. The pH values of water at a distance of (1–5) mm from the membrane surface are significantly nonlinear. The nonlinearity of the pH dependence near the membrane surface is observed both from time and from spatial coordinates and indicates the formation of concentration-capillary flows near the membrane surface. The pH gradient of distilled water reaches its maximum magnitude at a distance of 5 mm from the membrane surface and attains a peak value of approximately 0.6 mm^−1^; at 15 s. Nonequilibrium processes near the membrane surface stop after about (40–50) s, and, at the same time, the value of the pH does not change either in time or in the vicinity of the membrane.

Analysis of the IR spectra of membranes during their soaking in MB and AA solutions did not reveal new bands. The characteristic transmission minima of the membrane during its soaking in distilled water remain unchanged (Figure 6a) in the range of (1282–8000) nm. Analysis of the time-dependent transmission coefficients of the membrane (Figure 6b, Figure 7a,b and Figure 8) shows that the swelling time of the membrane in distilled water, aqueous solutions of Mb^+^, MbH^0^, and AA is 6, 8, 12, and 5 min, respectively.

The sorption rate of solutions into the membrane can be calculated using the formula:

(3) $$v=\frac{dK_{min}(t)}{dt},\;{min}^{-1}$$

Here, $K_{min}$ is the transmission coefficient of the membrane at the wavelength of ~1932 nm.

In distilled water and in MbH^0^ solution, the soaking rates are approximately the same. At the same time, the rate of soaking of Nafion in Mb^+^ solution is 75% higher than in MbH^0^ solution. There is no direct correlation between the results of IR spectrometry and the measurement of STC. However, the most significant changes occur in the IR spectrum and in the rheology of solutions over a time interval. The time dependences of the transmittance coefficient of a membrane with solutions in the IR range and the dependence of the STC are significantly nonlinear.

As the results of the absorption spectroscopy of the membrane and the Mb^+^ solution show (Figure 9), the adsorption of the MB solution into the membrane leads to a decrease in the concentration of the solution itself. This is evidenced by the decrease in the absorption coefficient of the solution. The current concentration of the solutions and the solution adsorbed in the membrane can be expressed through the absorption intensity:

(4) $$C\left(t\right)=\;C_{0}\frac{I(t)}{I_{0}},$$

where $C_{0}=C(t=0)$ is the initial concentration, and $I_{0}$ is the intensity of the characteristic spectral line at t=0, I(t) is the intensity of the spectral line at the current time. This spectral line can be selected for the Mb^+^ solution at the wavelength of the monomer.

During the drying of the Nafion membrane, water desorbs, and the adsorbed components of the solutions—MB and AA—remain on the membrane surface. The desorption of water leads to a redshift in the membrane’s absorption bands into the infrared region (Figure 9 and Figure 10). As a result, the membrane with the adsorbed components of the solutions forms its own absorption spectrum.

**Figure 14:**
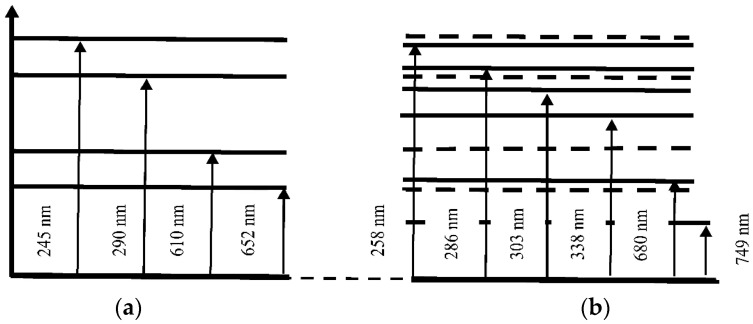
Schematic of the energy levels of the aqueous Mb^+^ solution (**a**) and the Nafion membrane soaked in the Mb^+^ solution (**b**).

**Figure 15:**
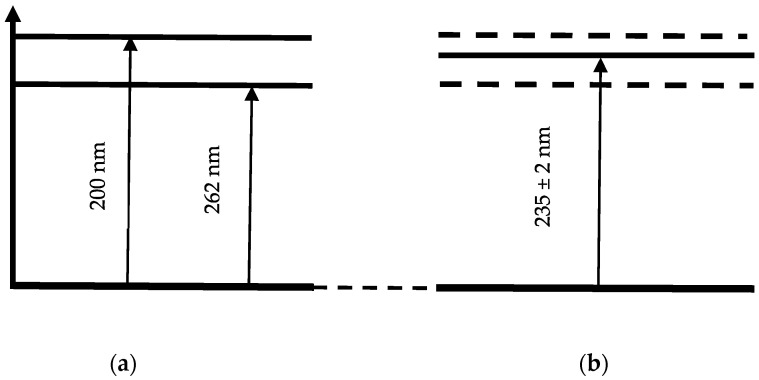
Schematic of the energy levels of the aqueous AA solution (**a**) and the Nafion membrane soaked in the AA solution (**b**).

Figure 14 and Figure 15 illustrate the possible electronic transitions that contribute to the absorption spectra of solutions and membranes containing Mb^+^ and AA adsorbents.

The energy levels corresponding to the absorption spectrum of an aqueous MB (Mb^+^) solution are shown in Figure 14a: 245, 290, 610, and 652 nm. Figure 14b shows the energy levels that correspond to the recorded absorption spectrum of the Nafion membrane after the absorption of an aqueous MB solution: 258, 286, 303, 338, 680 and 749 nm. The energy levels corresponding to the initial solution of MB (Mb^+^) are shown in Figure 14b with dashed lines. Two new spectral lines appear in the absorption spectrum of the MB-adsorbed Nafion membrane: at 303 and 338 nm. Additionally, a spectral doublet is formed at 286 and 303 nm (see Figure 9 and Figure 14). Concurrently, the other absorption bands of the “Nafion-MB” system undergo a redshift into the infrared region. The absorption bands at 680 and 749 nm in the “Nafion-MB” system result from the shift in the bands of the Mb^+^ solution, specifically from 610 and 652 nm, respectively. 

Figure 15a provides a schematic diagram of the energy levels corresponding to the absorption peaks of the aqueous AA solution at 200 and 262 nm. The energy levels corresponding to the absorption spectrum of the Nafion membrane after adsorption from an aqueous AA solution (band at 235 ± 2 nm) are shown in Figure 15b. The energy levels corresponding to the initial AA solution are shown by dashed lines in Figure 15b. Thus, a shift of the absorption lines is observed: for the Nafion membrane after MB solution adsorption, the shift is toward the IR region (Figure 9 and Figure 14), and for the Nafion membrane after aqueous AA solution adsorption, the shift is toward the UV region (Figure 10 and Figure 15). It can be assumed that the observed shift of the spectral bands is associated with distilled water desorption from the membrane. 

Let us consider the main factors and mechanisms influencing the adsorption and desorption processes of aqueous MB and AA solutions in the Nafion membrane. Let’s assume that the molecular complexes “Nafion-aqueous solutions” are located in a potential well [44,45]:

(5) Wx,t= −  Ax6+ Bx12−Ctexp−xRD

Here $R_{D}=\;\sqrt{\frac{\varepsilon \varepsilon_{0}kT}{8\pi e^{2}n_{i0}}}$—Debye screening radius [46]; *T*—the temperature; Ax6—the dispersion potential, where A—the dimensional constant; Bx12—the exchange repulsion potential, where *B* is the dimensional constant [36]; C(t) is a time—dependent dimensional constant; and x is the coordinate perpendicular to the membrane surface (*x* = 0 corresponds to the surface position) [45,47].

Assuming that at the initial time *t* = 0, which corresponds to the start of membrane soaking in the solution, in expression (5): $C(t=0)=C_{0}$. Coulomb forces dominate over the dispersion interaction in the system, which is associated with fluctuations in the dipole moments of particles [47]. The coordinate corresponding to the minimum of the Lennard-Jones potential W(x,0) for the particle complex under consideration can be found from the extremum condition of potential (5) at A = 0:

(6) $$x_{*}=\;\sqrt[{13}]{\frac{12BR_{D}}{C(0)}}$$

The depth of the potential well (5) and the coordinate of the potential minimum (6) depend on the value of the dielectric constant. The refractive index of an aqueous solution of AA with a concentration of 5 g/L has a value of 1.3335, MB solution—1.3470, and the distilled water used to prepare the solutions—1.3330. Therefore, the Debye screening length for Mb^+^ and AA will be greater than that for water. Then, the depth of the potential well will decrease, and the position of the potential minimum will increase. In work [42], the crystallization process of acids and salts from supersaturated aqueous solutions was monitored using refractive index measurements.

Compared to water molecules (molar mass 18 g/mol), MB and AA molecules are heavier species (molar mass of MB is 319.85 g/mol, AA is 176.12 g/mol). In this context, it can be assumed that MB and AA particles are located in potential wells and are practically immobile. Due to their significantly lower molar mass, water molecules undergo more active oscillatory motion compared to the heavier MB and AA molecules and, during membrane drying, rapidly desorb from the membrane, while the MB and AA molecules remain within the membrane and form a new absorption spectrum of the Nafion (Figure 9, Figure 10, Figure 14 and Figure 15).

Near the membrane surface, dissociation of sulfonic groups occurs. The negatively charged $SO_{3}^{-}$ groups [3] generate an electric field near the membrane’s planar surface, attracting protons and cations from the solution. Review [16] demonstrated that the electric field strength is close to that of a uniformly charged surface—$E_{0}(t)=\sigma (t)/(2\varepsilon \varepsilon_{0})$, where σ(t) is the surface charge density on the proton-exchange membrane due to the charge of the sulfonate groups upon dissociation, ε is the relative permittivity of distilled water, and $\varepsilon_{0}$ is the vacuum permittivity. Given the cationic nature of Mb^+^, it can be considered that this cation engages in Coulombic interaction with the sulfone groups ($SO_{3}^{-}$) of the membrane. In the case of Nafion membrane polymer fibrils unwound into the liquid volume, the electrically charged adsorbent surface is not planar, as the linear extent of the dissociation region of the charged polymer fibrils reaches 300 µm [15,16,48], which exceeds the Debye length.

Since the soaking of the membrane in solutions involves the diffusion of solution components into the membrane (Figure 9 and Figure 10), accompanied by a change in the surface tension of the solutions, it follows that concentration-capillary microflows can arise near the membrane surface. The magnitude of these concentration-capillary flows generated near the membrane surface can be estimated from the results of the solution surface tension coefficient measurements. A time interval of 10 min was selected to evaluate the surface driving force. From Figure 3, Figure 4 and Figure 5, it can be seen that the maximum changes in STC value are observed for the MBH^0^ solution, as its STC changes over this time interval are the greatest. According to the estimation, the surface force density near the membrane surface for the Mb^+^ solution at a distance of 100 µm from the membrane surface is ~46 N/m^2^, for the AA solution ~60 N/m^2^, and for the MBH^0^ solution ~130 N/m^2^.

Thus, the presence of a Nafion membrane affects the rheology of aqueous solutions and the rate range of cation diffusion process into the membrane from aqueous solutions. This explains the specifics of the crystallization from aqueous solutions of salts and acids in the presence of an ion-exchange membrane. For example, the results of research on the crystallization of salts and acids from supersaturated aqueous solutions on a polymer substrate of the Nafion membrane are presented in [42,43]. It was determined that, in a supersaturated aqueous solution prepared on the basis of DDW (3 ppm), copper sulfate pentahydrate CuSO_4_ · 5H_2_O with triclinic syngony grows on the membrane surface. The authors of [42] suggest that, when using a supersaturated solution prepared using MQ, the degree of dissociation of sulfonic groups increases, and for this reason crystals of copper sulfate trihydrate (CuSO_4_ · 3H_2_O, monoclinic syngony) are formed on the polymer substrate. The supersaturated aqueous solution of ASC does not crystallize in the presence of the Nafion membrane but acquires an amorphous state. Changes in the coefficient of surface tension of aqueous solutions of salts and acids near the surface of the ion exchange membrane will affect the size of the nucleation during crystallization.

On the one hand, fuel cells with a proton exchange membrane are their direct and most common technical application [49,50]. An urgent task is still to improve the properties of membranes in order to increase the efficiency of hydrogen fuel cells. The review [51] describes in detail the latest achievements in the field of modification of membranes made of perfluorinated compounds in order to increase the service life of membranes and increase their efficiency. To solve these problems, organic–inorganic hybrids are being introduced, and partially fluorinated and non-fluorinated membranes are being used to increase the efficiency of perfluorinated compounds in fuel cells with proton exchange membranes.

There are various dopants for creating hybrid membranes based on the Nafion membrane. Modification of the Nafion membrane with graphene oxide makes it possible to improve the characteristics of vanadium redox batteries [52]. To improve proton conductivity and achieve maximum output power, which is higher than that of commercial Nafion, Nafion membranes are manufactured by casting, and aryl- or azaheteroaromatic bisphosphonate compounds are used as dopant modifiers [53]. For example, by impregnating the Nafion membrane, silica nanocomposite membranes are obtained based on Nafion [54].

To increase the mechanical strength and proton conductivity in fuel cells, the Nafion membrane is reinforced with nanofibers, and graphene-based films are applied to the Nafion [51,55]. Graphene acts as a selective diffusion barrier, which increases the stability of cation exchange layers and, in general, the operational stability of fuel cells. Hybrid Nafion membranes are produced by filling membranes with zirconium dioxide nanoparticles, which are characterized by moisture content, proton conductivity, and heat resistance [56], and membranes with inorganic fillers, carbon nanomaterials, and ionic liquids are being developed to increase proton conductivity and regulate membrane permeability to methanol [57].

On the other hand, the field of application of ion exchange membranes is expanding. Ion exchange membranes are used not only in energy but also in ecology and medicine. Membrane technologies enable the solution of tasks related to solution separation and purification for ensuring environmental safety, enhance the stability of cation-exchange layers for conducting electrodialysis [58], and improve the energy efficiency of technological processes [49,50]. To optimize the control of fuel cells with a proton exchange membrane and the efficient operation of micro-mixers on ion exchange membranes, it is necessary to consider concentration-capillary and thermo-capillary convection.

The results of experiments on measuring the STC of solutions in the presence of a membrane and spectrophotometry indicate that the kinetics of formation of an exclusion zone near the membrane depend on the type of solution. Concentration-capillary flows occur together with the formation of the excluded zone. Their intensity is determined by the value of the gradient of the STC of the solution and its concentration. Such flows occur both near the membrane surface and in its volume. The intensity of concentration-capillary flows in the considered areas is different: it is significantly higher near the membrane surface than in its volume. From experiments on soaking the membrane in MB solution (Figure 12), it turns out that the diffusion rate into the membrane of MB solution prepared in distilled water is 28 × 10^−3^ min^−1^. Based on the results of absorption spectrophotometry experiments, when the Nafion membrane is soaked in distilled water (157 ppm), the swelling time of the membrane is approximately (60–70) minutes (Figure 11). At the same time, the time interval in which intensive changes in the STC of solutions are observed is, on average, about 10 min for the studied aqueous solutions and MQ (Figure 2, Figure 3, Figure 4 and Figure 5). Therefore, it can be assumed that the flows in the near-surface region into the volume of the solution will be about 7 times greater than into the volume of the membrane. The intensity of the liquid flows near the membrane surface decreases as the membrane swells. The creation of additional forces in the volume of liquid near the membrane and in the volume of the membrane is relevant for systems containing ion-exchange membranes, during the adsorption and desorption of solutions required for the extraction of components, during the transportation of medicinal components of solutions in biological systems.

Also, the creation of the «Nafion-MB» and «Nafion-AA» light filters is a practically useful result of this research. Such filters, in the range of (190–900) nm, have additional absorption lines that differ from the absorption lines of the neat (untreated) Nafion and an aqueous solution of Mb^+^ [35,36]. The kinetics of the surface tension coefficient of aqueous solutions of MB and AA, observed in the presence of a Nafion membrane, qualitatively correlate with the kinetics of the transmission spectrum of aqueous solutions of MB and AA, detected at a wavelength of 1932 nm. Additional concentration-capillary flows occur near the membrane surface during dissociation of sulfogroups. In the presence of components of the solution that increase its conductivity, we get the opportunity to control redox processes in an aqueous solution without additional energy consumption in the form of heat or ultrasound exposure. The appearance of slow diffusion flows inside the membrane volume indicates the possibility of controlling redox reactions in the volume of the membrane itself, and not only in solution. Our preliminary experiments indicate the possibility of a redox reaction both in the solution surrounding the membrane and inside the membrane itself. That is, it is possible to use the membrane as a medium in which local chemical reactions can be carried out.

Really, in this work we have tried to supplement the information about the rheological properties of water and aqueous solutions, in particular, organic compounds in the presence of a proton exchange membrane Nafion. The membrane, reacting to the cations of solutions due to the dissociation of sulfogroups in them, creates additional ion movements near the surface. For example, when crystals are grown on a polymer substrate made of Nafion, the effect of the membrane on the surface tension coefficient of the solution will determine the crystal growth rate and their size. It is known that the size of the crystallization embryo is determined by the balance of volumetric and surface energies. An increase or decrease in the STC value of the solution will determine the size of the embryo during crystallization.

Based on the results of experiments on IR-Fourier spectroscopy (Figure 6, Figure 7 and Figure 8), absorption spectroscopy (Figure 9 and Figure 10), we suggest that the formation of the excluded zone near the membrane surface depends on the type of solution. Concentration-capillary flows occur together with the formation of the excluded zone. Their intensity is determined by the value of the gradient of the STC of the solution and its concentration. Concentration-capillary flows occur both near the membrane surface and in its volume. The intensity of concentration-capillary flows in the considered areas is different. It is significantly larger near the membrane surface than in its volume. Based on the results of absorption spectrophotometry experiments, when the Nafion membrane is soaked in distilled water (157 ppm), the swelling time of the membrane is approximately 70 min (Figure 11). To illustrate the processes occurring in the membrane, we have added Figure 11 and Figure 12 to the manuscript. At the same time, the time interval in which an intensive change in the STC of solutions is observed is, on average, about 10 min for the studied aqueous solutions and MQ (Figure 2, Figure 3, Figure 4 and Figure 5). Therefore, it can be assumed that the flows in the near-surface area in the volume of the solution will be about 7 times greater than in the volume of the membrane. As shown by the time dependences of the STC of solutions during membrane soaking, the flows near the surface decrease in intensity as the membrane swells. The creation of additional forces in the volume of liquid near the membrane and in the volume of the membrane is relevant for systems containing ion-exchange membranes, during the adsorption and desorption of solutions required for the extraction of components, during the transportation of medicinal components of solutions in biological systems.

## 5. Conclusions

The time dependence of the surface tension coefficient for aqueous solutions of various forms of methylene blue and ascorbic acid in the presence of a Nafion membrane is substantially nonlinear. On the one hand, the kinetics of the surface tension coefficient of solutions during a membrane soaking are associated with the processes of diffusion of Mb^+^ and MbH^0^ onto the membrane surface. On the other hand, studies show [11] that the pH value of distilled water at a distance of 1 mm from the membrane surface decreases by about 54% after 15 s of soaking in water. The time dependence of the pH of distilled water in the presence of a membrane is also a nonlinear function, and 50 s after the start of soaking, the pH value of distilled water at the same distance from the membrane surface increases, with the deviation from the initial water pH value then being only 15%. It follows that the size of the exclusive zone near the membrane surface should depend significantly on both the type of solution and the composition of the aqueous medium.

The process of exclusion zone formation near the membrane surface is accompanied by the emergence of a pH gradient [11] and an STC gradient. The presence of the STC gradient will induce additional concentration-capillary flows in the solution, which form within the exclusion zone region. The influence of the Nafion membrane on the rheological properties of solutions will, in turn, affect the kinetics of the reduction reaction of the aqueous Mb^+^ solution, implemented using the ascorbic acid solution.

## Data Availability

The original contributions presented in this study are included in the article. Further inquiries can be directed to the corresponding author.
